# Crystal structure of hexa­kis­(dimethyl sulfoxide-κ*O*)manganese(II) diiodide

**DOI:** 10.1107/S2056989016008896

**Published:** 2016-06-10

**Authors:** Mathias Glatz, Martina Schroffenegger, Matthias Weil, Karl Kirchner

**Affiliations:** a Institute of Applied Synthetic Chemistry, Vienna University of Technology, Getreidemarkt 9/163, A-1060 Vienna, Austria; b Institute for Chemical Technologies and Analytics, Division of Structural Chemistry, Vienna University of Technology, Getreidemarkt 9/164-SC, A-1060 Vienna, Austria

**Keywords:** crystal structure, dimethyl sulfoxide, manganese(II), octa­hedral coordination

## Abstract

The asymmetric unit of the title salt, [Mn(C_2_H_6_OS)_6_]I_2_, consists of one Mn^II^ ion, six O-bound dimethyl sulfoxide (DMSO) ligands and two I^−^ counter-anions. The isolated complex cations have an octa­hedral configuration and are grouped in hexa­gonally arranged rows extending parallel to [100]. The two I^−^ anions are located between the rows and are linked to the cations through two weak C—H⋯I inter­actions.

## Abstract 2

The title salt consists of isolated octa­hedrally shaped [Mn(DMSO)_6_]^2+^ cations (DMSO is dimethyl sulfoxide) and two I^−^ anions, held together through weak C—H⋯I inter­actions.

## Chemical context   

**Figure 1:**
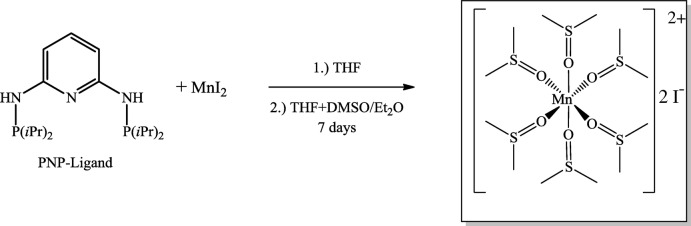
Schematic representation of the attempted formation of a manganese(II) complex with the PNP ligand.

Tridentate pincer ligands coordinating either through two P and one N atom (PNP-type) or through two P and one C atom (PCP-type) have multifarious applications in catalysis, synthetic chemistry or mol­ecular recognition (Szabo & Wendt, 2014 ▸). Although these ligands play an important role in coordination chemistry, studies of pincer complexes of first-row transition metals are rather scarce (Murugesan & Kirchner, 2016 ▸). During a current project to prepare the first manganese(II) PNP-type pincer complexes (Mastalir *et al.*, 2016 ▸) according to the reaction scheme presented in Fig. 1 ▸, we obtained instead the title salt, [Mn(DMSO)_6_]I_2_ (DMSO is dimethyl sulfoxide), and report here its crystal structure.
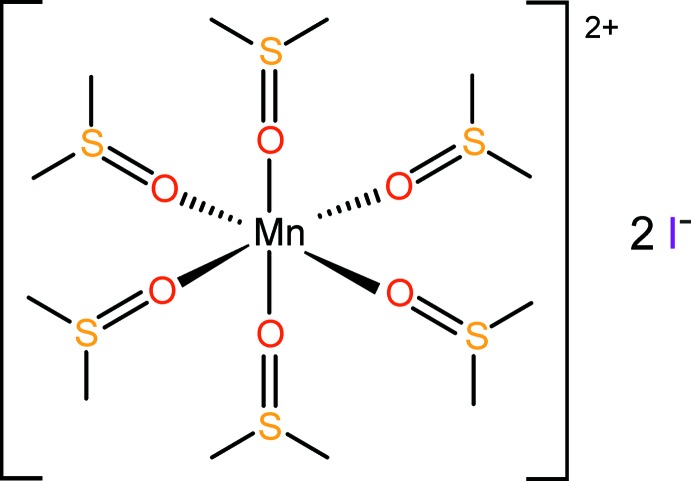


## Structural commentary   

**Figure 2:**
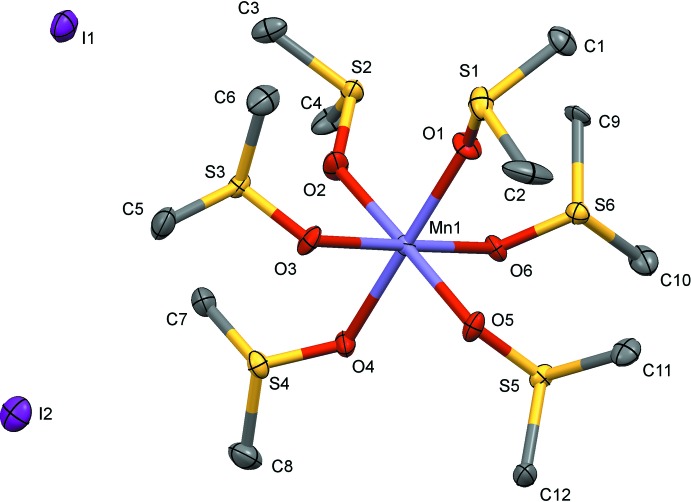
The structures of the mol­ecular and ionic entities in the title salt, showing the atom labelling. Displacement ellipsoids are drawn at the 50% probability level and, for clarity, the H atoms have been omitted.

The Mn^2+^ cation is bound to the O atoms of six DMSO mol­ecules that are arranged in an octa­hedral configuration around the metal cation (Fig. 2 ▸). The deviation from the ideal octa­hedral coordination are minute, with *cis* O—Mn—O angles ranging from 85.8 (2) to 93.8 (2)° and *trans* angles from 176.3 (2) to 178.2 (2)°. The averaged Mn—O bond length of 2.17 (2) Å is in perfect agreement with that of the related perchlorate salt [Mn(DMSO)_6_](ClO_4_)_2_ [2.167 (14) Å; Migdał-Mikuli *et al.*, 2006 ▸] that also consists of isolated [Mn(DMSO)_6_]^2+^ cations and non-coordinating anions.

## Supra­molecular features   

**Figure 3:**
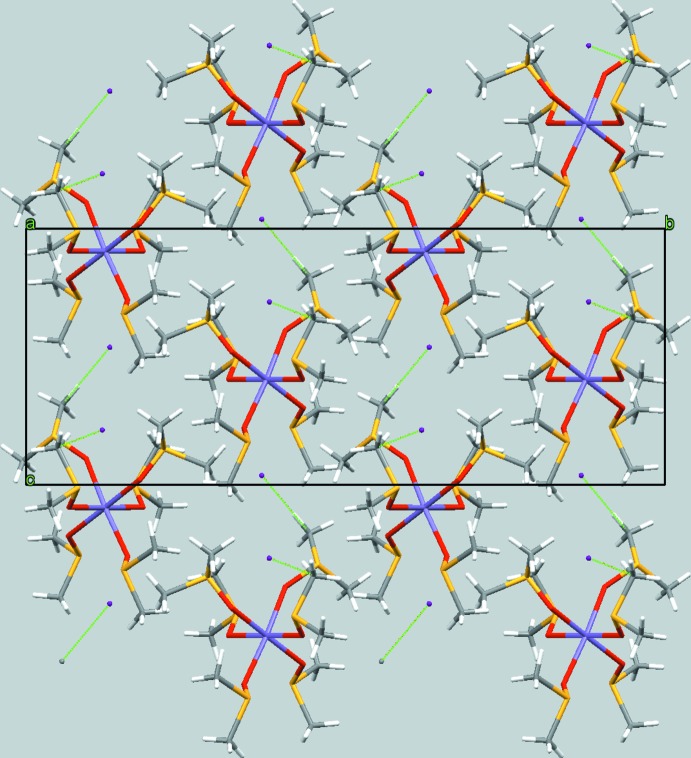
A projection of the crystal structure along [100], showing the stacking of the complex cations of the title salt in this direction. C—H⋯I interactions are shown as green dashed lines.

The isolated complex [Mn(DMSO)_6_]^2+^ mol­ecules are stacked into rows extending parallel to [100] whereby the rows are arranged in a distorted hexa­gonal rod packing. The iodide counter-anions are located between the rows and, apart from Coulomb inter­actions, are linked to the complex cations through weak C—H⋯I inter­actions (Table 1 ▸, Fig. 3 ▸).

## Database survey   

A search in the Cambridge Structural Database (Groom *et al.*, 2016 ▸) for structures of divalent metal compounds containing octa­hedrally shaped [*M*(DMSO]^2+^ cations (*M* = Mg, Mn, Fe, Co, Ni, Cu, Zn, Cd, Hg) revealed 50 entries. From these, only four were manganese compounds. A number of iodine-containing structures are also included in this hit list, but these structures either contain polyiodide anions (I_3_^−^ or I_4_^2−^) or complex anions of the type [*M*I_4_]^2−^. Therefore, the title compound is the first compound with [*M*(DMSO]^2+^ cations and simple iodide anions.

## Synthesis and crystallization   

All manipulations were performed under an inert atmosphere of argon by using Schlenk techniques or in a MBraun inert-gas glove box. The solvents were purified according to standard procedures. Anhydrous MnI_2_ was purchased from Sigma–Aldrich and was used without further purification. The synthesis of the PNP-ligand was performed according to literature procedures (Benito-Garagorri *et al.*, 2006 ▸).

The title manganese salt was formed in the course of the targeted synthesis of an Mn^II^ PNP-complex (Fig. 1 ▸). Anhydrous MnI_2_ (93 mg, 0.50 mmol) and the PNP-ligand (115 mg, 0.33 mmol) were stirred in 7 ml tetra­hydro­furan for one h. 2 ml of DMSO were added and the solution filtrated over celite. The clear colourless solution was layered with 15 ml diethyl ether and was left for 7 days. Colourless crystals of the title compound were obtained as the only solid reaction product.

## Refinement   

Crystal data, data collection and structure refinement details are summarized in Table 2 ▸. Close inspection of the diffraction pattern revealed twinning by non-merohedry with one domain rotated by 180° about [100]. Intensity statistics showed 1583 reflections belonging to domain 1 only (mean *I*/*σ* = 7.5), 1583 reflections to domain 2 only (mean *I*/*σ* = 7.2) and 4780 reflections to both domains (mean *I*/*σ* = 7.5). The presence of two domains with equal scattering volume was confirmed by the refinement (refinement as a two-component twin using an HKLF-5 file). The refined Flack parameter (Flack, 1983 ▸) of 0.10 (2) revealed additional twinning by inversion. The maximum remaining electron density is found 1.30 Å from atom H2*C* and the minimum remaining electron density 1.06 Å from atom I1.

## Supplementary Material

Crystal structure: contains datablock(s) I, global. DOI: 10.1107/S2056989016008896/su5305sup1.cif

Structure factors: contains datablock(s) I. DOI: 10.1107/S2056989016008896/su5305Isup2.hkl

CCDC reference: 1483114

Additional supporting information: crystallographic information; 3D view; checkCIF report

## Figures and Tables

**Table 1:** Hydrogen-bond geometry (Å, °)

*D*—H⋯*A*	*D*—H	H⋯*A*	*D*⋯*A*	*D*—H⋯*A*
C1—H1*C*⋯I2^i^	0.98	3.03	3.926 (10)	152
C6—H6*B*⋯I1	0.98	3.05	3.878 (12)	143

Symmetry code: (i) 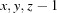.

**Table 2:** Experimental details

Crystal data	
Chemical formula	[Mn(C_2_H_6_OS)_6_]I_2_
*M*_r_	777.51
Crystal system, space group	Monoclinic, *C* *c*
Temperature (K)	100
*a*, *b*, *c* (Å)	12.0996 (14), 24.511 (3), 11.2999 (13)
β (°)	119.577 (3)
*V* (Å^3^)	2914.6 (6)
*Z*	4
Radiation type	Mo *K*α
μ (mm^−1^)	3.02
Crystal size (mm)	0.15 × 0.10 × 0.05

Data collection	
Diffractometer	Bruker APEXII CCD
Absorption correction	Multi-scan (*TWINABS*; Bruker, 2014 ▸)
*T*_min_, *T*_max_	0.574, 0.746
No. of measured, independent and observed [*I* > 2σ(*I*)] reflections	4935, 4935, 4279
(sin θ/λ)_max_ (Å^−1^)	0.743

Refinement	
*R*[*F* ^2^ > 2σ(*F* ^2^)], *wR*(*F* ^2^), *S*	0.044, 0.074, 1.16
No. of reflections	4935
No. of parameters	257
No. of restraints	2
H-atom treatment	H-atom parameters constrained
Δρ_max_, Δρ_min_ (e Å^−3^)	2.11, −1.51
Absolute structure	No quotients, so Flack parameter determined by classical intensity fit
Absolute structure parameter	0.10 (2)

Computer programs: *APEX2* and *SAINT-Plus* (Bruker, 2014 ▸), *SHELXS97* (Sheldrick, 2008 ▸), *SHELXL2014* (Sheldrick, 2015 ▸), *Mercury* (Macrae *et al.*, 2006 ▸) and *publCIF* (Westrip, 2010 ▸).

## supplementary crystallographic information

### Crystal data

**Table:**

[Mn(C_2_H_6_OS)_6_]I_2_	*F*(000) = 1532
*M**_r_* = 777.51	*D*_x_ = 1.772 Mg m^−^^3^
Monoclinic, *C**c*	Mo *K*α radiation, λ = 0.71073 Å
*a* = 12.0996 (14) Å	Cell parameters from 9642 reflections
*b* = 24.511 (3) Å	θ = 2.2–31.3°
*c* = 11.2999 (13) Å	µ = 3.02 mm^−^^1^
β = 119.577 (3)°	*T* = 100 K
*V* = 2914.6 (6) Å^3^	Fragment, colourless
*Z* = 4	0.15 × 0.10 × 0.05 mm

### Data collection

**Table:**

Bruker APEXII CCD diffractometer	4279 reflections with *I* > 2σ(*I*)
ω– and φ–scans	θ_max_ = 31.9°, θ_min_ = 1.7°
Absorption correction: multi-scan (*TWINABS*; Bruker, 2014)	*h* = −17→15
*T*_min_ = 0.574, *T*_max_ = 0.746	*k* = 0→35
4935 measured reflections	*l* = 0→16
4935 independent reflections	

### Refinement

**Table:**

Refinement on *F*^2^	Hydrogen site location: inferred from neighbouring sites
Least-squares matrix: full	H-atom parameters constrained
*R*[*F*^2^ > 2σ(*F*^2^)] = 0.044	*w* = 1/[σ^2^(*F*_o_^2^) + (0.0202*P*)^2^ + 8.7709*P*] where *P* = (*F*_o_^2^ + 2*F*_c_^2^)/3
*wR*(*F*^2^) = 0.074	(Δ/σ)_max_ = 0.001
*S* = 1.16	Δρ_max_ = 2.11 e Å^−^^3^
4935 reflections	Δρ_min_ = −1.51 e Å^−^^3^
257 parameters	Absolute structure: No quotients, so Flack parameter determined by classical intensity fit
2 restraints	Absolute structure parameter: 0.10 (2)

### Special details

**Table:**

Geometry. All esds (except the esd in the dihedral angle between two l.s. planes) are estimated using the full covariance matrix. The cell esds are taken into account individually in the estimation of esds in distances, angles and torsion angles; correlations between esds in cell parameters are only used when they are defined by crystal symmetry. An approximate (isotropic) treatment of cell esds is used for estimating esds involving l.s. planes.
Refinement. Refined as a 2-component twin.

### Fractional atomic coordinates and isotropic or equivalent isotropic displacement parameters (Å^2^)

**Table:**

	*x*	*y*	*z*	*U*_iso_*/*U*_eq_	
Mn1	0.92250 (11)	0.87649 (5)	0.58801 (9)	0.0112 (2)	
I1	0.26974 (6)	0.88077 (3)	0.28565 (7)	0.02714 (14)	
I2	0.58683 (5)	0.86891 (2)	0.96305 (5)	0.02930 (16)	
S1	0.8652 (2)	0.95666 (9)	0.3103 (3)	0.0250 (5)	
S2	0.7237 (2)	0.78418 (9)	0.3582 (2)	0.0186 (4)	
S4	0.8551 (2)	0.84771 (8)	0.8308 (2)	0.0180 (4)	
S3	0.64117 (19)	0.91912 (7)	0.5116 (2)	0.0151 (4)	
S5	1.2168 (2)	0.91633 (8)	0.7738 (2)	0.0159 (4)	
S6	1.12193 (18)	0.83029 (8)	0.5061 (2)	0.0155 (4)	
O1	0.8988 (6)	0.9059 (2)	0.3974 (6)	0.0223 (13)	
O2	0.7667 (6)	0.8219 (2)	0.4797 (6)	0.0229 (13)	
O3	0.7823 (6)	0.9325 (2)	0.5874 (7)	0.0199 (12)	
O4	0.9477 (5)	0.8411 (2)	0.7784 (6)	0.0188 (11)	
O5	1.0771 (6)	0.9339 (2)	0.6990 (6)	0.0181 (12)	
O6	1.0538 (6)	0.8170 (2)	0.5873 (6)	0.0165 (11)	
C1	0.9114 (10)	0.9439 (4)	0.1885 (9)	0.025 (2)	
H1A	0.9949	0.9261	0.2322	0.037*	
H1B	0.9163	0.9784	0.1476	0.037*	
H1C	0.8489	0.9199	0.1176	0.037*	
C2	0.9819 (13)	1.0048 (4)	0.4068 (10)	0.045 (3)	
H2A	0.9720	1.0163	0.4842	0.067*	
H2B	0.9729	1.0366	0.3500	0.067*	
H2C	1.0662	0.9886	0.4404	0.067*	
C3	0.5563 (9)	0.7902 (4)	0.2691 (10)	0.025 (2)	
H3A	0.5253	0.7927	0.3343	0.037*	
H3B	0.5190	0.7581	0.2111	0.037*	
H3C	0.5317	0.8231	0.2126	0.037*	
C4	0.7368 (10)	0.7169 (3)	0.4265 (11)	0.029 (2)	
H4A	0.8266	0.7063	0.4775	0.043*	
H4B	0.6904	0.6910	0.3519	0.043*	
H4C	0.7008	0.7166	0.4874	0.043*	
C5	0.5721 (9)	0.9626 (4)	0.5828 (11)	0.030 (2)	
H5A	0.5888	0.9480	0.6708	0.045*	
H5B	0.4801	0.9647	0.5212	0.045*	
H5C	0.6091	0.9992	0.5956	0.045*	
C6	0.5831 (11)	0.9507 (4)	0.3532 (11)	0.030 (2)	
H6A	0.6037	0.9896	0.3663	0.045*	
H6B	0.4907	0.9460	0.3004	0.045*	
H6C	0.6224	0.9340	0.3041	0.045*	
C7	0.7344 (8)	0.7981 (4)	0.7471 (9)	0.027 (2)	
H7A	0.6856	0.8066	0.6498	0.041*	
H7B	0.6776	0.7983	0.7859	0.041*	
H7C	0.7732	0.7619	0.7594	0.041*	
C8	0.9331 (10)	0.8176 (4)	0.9972 (10)	0.032 (2)	
H8A	0.9538	0.7794	0.9905	0.049*	
H8B	0.8767	0.8191	1.0363	0.049*	
H8C	1.0115	0.8377	1.0558	0.049*	
C9	1.0351 (9)	0.7942 (3)	0.3481 (8)	0.0189 (15)	
H9A	1.0336	0.7552	0.3664	0.028*	
H9B	1.0763	0.7996	0.2930	0.028*	
H9C	0.9478	0.8081	0.2986	0.028*	
C10	1.2612 (10)	0.7887 (4)	0.5827 (10)	0.027 (2)	
H10A	1.3156	0.7999	0.6775	0.040*	
H10B	1.3077	0.7929	0.5327	0.040*	
H10C	1.2367	0.7504	0.5798	0.040*	
C11	1.2929 (9)	0.9566 (4)	0.7044 (10)	0.024 (2)	
H11A	1.2599	0.9468	0.6087	0.036*	
H11B	1.3847	0.9500	0.7553	0.036*	
H11C	1.2761	0.9953	0.7109	0.036*	
C12	1.2843 (9)	0.9470 (4)	0.9385 (9)	0.0231 (19)	
H12A	1.2857	0.9868	0.9295	0.035*	
H12B	1.3713	0.9336	0.9954	0.035*	
H12C	1.2330	0.9375	0.9807	0.035*	

### Atomic displacement parameters (Å^2^)

**Table:**

	*U*^11^	*U*^22^	*U*^33^	*U*^12^	*U*^13^	*U*^23^
Mn1	0.0126 (5)	0.0097 (5)	0.0119 (5)	−0.0014 (5)	0.0064 (5)	0.0008 (5)
I1	0.0220 (3)	0.0280 (3)	0.0317 (3)	−0.0063 (3)	0.0135 (3)	0.0007 (3)
I2	0.0279 (3)	0.0216 (3)	0.0371 (4)	0.0059 (3)	0.0150 (3)	0.0006 (3)
S1	0.0234 (11)	0.0249 (11)	0.0318 (13)	0.0063 (9)	0.0176 (10)	0.0097 (9)
S2	0.0166 (9)	0.0199 (9)	0.0201 (10)	−0.0018 (8)	0.0096 (8)	−0.0062 (8)
S4	0.0225 (10)	0.0153 (8)	0.0214 (10)	−0.0009 (7)	0.0148 (8)	0.0014 (7)
S3	0.0109 (9)	0.0130 (8)	0.0188 (9)	0.0001 (7)	0.0053 (8)	−0.0004 (7)
S5	0.0146 (10)	0.0118 (8)	0.0188 (9)	−0.0012 (7)	0.0064 (8)	0.0000 (8)
S6	0.0169 (10)	0.0144 (8)	0.0154 (9)	0.0007 (7)	0.0082 (7)	−0.0008 (7)
O1	0.031 (3)	0.021 (3)	0.019 (3)	0.001 (3)	0.015 (3)	0.010 (2)
O2	0.024 (3)	0.021 (3)	0.028 (3)	−0.010 (2)	0.016 (3)	−0.011 (2)
O3	0.010 (3)	0.016 (3)	0.028 (3)	0.004 (2)	0.006 (3)	−0.002 (2)
O4	0.016 (3)	0.026 (3)	0.019 (3)	0.004 (2)	0.011 (2)	0.006 (2)
O5	0.012 (3)	0.013 (3)	0.028 (3)	−0.001 (2)	0.009 (3)	−0.001 (2)
O6	0.022 (3)	0.015 (3)	0.018 (3)	0.000 (2)	0.015 (2)	0.000 (2)
C1	0.033 (5)	0.023 (4)	0.024 (5)	−0.004 (4)	0.019 (4)	0.003 (4)
C2	0.075 (9)	0.037 (5)	0.015 (5)	−0.021 (6)	0.016 (5)	−0.002 (4)
C3	0.024 (5)	0.017 (4)	0.023 (4)	0.002 (4)	0.004 (4)	−0.003 (3)
C4	0.022 (5)	0.016 (4)	0.031 (6)	0.005 (4)	0.000 (4)	0.004 (4)
C5	0.016 (4)	0.039 (5)	0.036 (6)	−0.003 (4)	0.012 (4)	−0.008 (5)
C6	0.027 (5)	0.029 (5)	0.028 (5)	−0.005 (4)	0.009 (5)	0.011 (4)
C7	0.024 (5)	0.040 (5)	0.024 (5)	−0.004 (4)	0.016 (4)	−0.003 (3)
C8	0.040 (6)	0.038 (5)	0.021 (5)	−0.004 (4)	0.017 (4)	−0.004 (4)
C9	0.025 (4)	0.022 (4)	0.013 (4)	−0.002 (4)	0.012 (4)	−0.008 (3)
C10	0.029 (5)	0.030 (5)	0.026 (5)	0.010 (4)	0.016 (4)	0.009 (4)
C11	0.020 (5)	0.022 (4)	0.024 (5)	0.004 (4)	0.007 (4)	0.008 (4)
C12	0.017 (4)	0.033 (5)	0.020 (4)	−0.006 (4)	0.010 (4)	−0.008 (4)

### Geometric parameters (Å, º)

**Table:**

Mn1—O2	2.137 (6)	C3—H3A	0.9800
Mn1—O1	2.152 (6)	C3—H3B	0.9800
Mn1—O6	2.159 (6)	C3—H3C	0.9800
Mn1—O5	2.176 (6)	C4—H4A	0.9800
Mn1—O3	2.180 (6)	C4—H4B	0.9800
Mn1—O4	2.197 (6)	C4—H4C	0.9800
S1—O1	1.512 (6)	C5—H5A	0.9800
S1—C2	1.749 (11)	C5—H5B	0.9800
S1—C1	1.751 (10)	C5—H5C	0.9800
S2—O2	1.518 (6)	C6—H6A	0.9800
S2—C3	1.768 (10)	C6—H6B	0.9800
S2—C4	1.795 (9)	C6—H6C	0.9800
S4—O4	1.512 (6)	C7—H7A	0.9800
S4—C7	1.773 (9)	C7—H7B	0.9800
S4—C8	1.795 (10)	C7—H7C	0.9800
S3—O3	1.521 (6)	C8—H8A	0.9800
S3—C6	1.747 (10)	C8—H8B	0.9800
S3—C5	1.774 (10)	C8—H8C	0.9800
S5—O5	1.532 (6)	C9—H9A	0.9800
S5—C11	1.775 (10)	C9—H9B	0.9800
S5—C12	1.787 (9)	C9—H9C	0.9800
S6—O6	1.541 (6)	C10—H10A	0.9800
S6—C10	1.786 (9)	C10—H10B	0.9800
S6—C9	1.795 (8)	C10—H10C	0.9800
C1—H1A	0.9800	C11—H11A	0.9800
C1—H1B	0.9800	C11—H11B	0.9800
C1—H1C	0.9800	C11—H11C	0.9800
C2—H2A	0.9800	C12—H12A	0.9800
C2—H2B	0.9800	C12—H12B	0.9800
C2—H2C	0.9800	C12—H12C	0.9800
			
O2—Mn1—O1	89.6 (2)	H3B—C3—H3C	109.5
O2—Mn1—O6	91.0 (2)	S2—C4—H4A	109.5
O1—Mn1—O6	87.6 (2)	S2—C4—H4B	109.5
O2—Mn1—O5	178.2 (2)	H4A—C4—H4B	109.5
O1—Mn1—O5	90.7 (2)	S2—C4—H4C	109.5
O6—Mn1—O5	90.8 (2)	H4A—C4—H4C	109.5
O2—Mn1—O3	85.8 (2)	H4B—C4—H4C	109.5
O1—Mn1—O3	93.8 (2)	S3—C5—H5A	109.5
O6—Mn1—O3	176.5 (2)	S3—C5—H5B	109.5
O5—Mn1—O3	92.3 (2)	H5A—C5—H5B	109.5
O2—Mn1—O4	88.3 (2)	S3—C5—H5C	109.5
O1—Mn1—O4	176.3 (2)	H5A—C5—H5C	109.5
O6—Mn1—O4	89.4 (2)	H5B—C5—H5C	109.5
O5—Mn1—O4	91.5 (2)	S3—C6—H6A	109.5
O3—Mn1—O4	89.1 (2)	S3—C6—H6B	109.5
O1—S1—C2	106.0 (4)	H6A—C6—H6B	109.5
O1—S1—C1	106.0 (4)	S3—C6—H6C	109.5
C2—S1—C1	97.9 (6)	H6A—C6—H6C	109.5
O2—S2—C3	104.4 (4)	H6B—C6—H6C	109.5
O2—S2—C4	104.7 (4)	S4—C7—H7A	109.5
C3—S2—C4	98.9 (5)	S4—C7—H7B	109.5
O4—S4—C7	107.0 (4)	H7A—C7—H7B	109.5
O4—S4—C8	105.0 (4)	S4—C7—H7C	109.5
C7—S4—C8	98.3 (5)	H7A—C7—H7C	109.5
O3—S3—C6	104.7 (5)	H7B—C7—H7C	109.5
O3—S3—C5	105.3 (4)	S4—C8—H8A	109.5
C6—S3—C5	99.0 (5)	S4—C8—H8B	109.5
O5—S5—C11	105.8 (4)	H8A—C8—H8B	109.5
O5—S5—C12	105.6 (4)	S4—C8—H8C	109.5
C11—S5—C12	98.9 (5)	H8A—C8—H8C	109.5
O6—S6—C10	104.2 (4)	H8B—C8—H8C	109.5
O6—S6—C9	105.4 (4)	S6—C9—H9A	109.5
C10—S6—C9	98.6 (5)	S6—C9—H9B	109.5
S1—O1—Mn1	141.9 (4)	H9A—C9—H9B	109.5
S2—O2—Mn1	135.3 (4)	S6—C9—H9C	109.5
S3—O3—Mn1	121.5 (3)	H9A—C9—H9C	109.5
S4—O4—Mn1	124.7 (3)	H9B—C9—H9C	109.5
S5—O5—Mn1	122.4 (3)	S6—C10—H10A	109.5
S6—O6—Mn1	118.1 (3)	S6—C10—H10B	109.5
S1—C1—H1A	109.5	H10A—C10—H10B	109.5
S1—C1—H1B	109.5	S6—C10—H10C	109.5
H1A—C1—H1B	109.5	H10A—C10—H10C	109.5
S1—C1—H1C	109.5	H10B—C10—H10C	109.5
H1A—C1—H1C	109.5	S5—C11—H11A	109.5
H1B—C1—H1C	109.5	S5—C11—H11B	109.5
S1—C2—H2A	109.5	H11A—C11—H11B	109.5
S1—C2—H2B	109.5	S5—C11—H11C	109.5
H2A—C2—H2B	109.5	H11A—C11—H11C	109.5
S1—C2—H2C	109.5	H11B—C11—H11C	109.5
H2A—C2—H2C	109.5	S5—C12—H12A	109.5
H2B—C2—H2C	109.5	S5—C12—H12B	109.5
S2—C3—H3A	109.5	H12A—C12—H12B	109.5
S2—C3—H3B	109.5	S5—C12—H12C	109.5
H3A—C3—H3B	109.5	H12A—C12—H12C	109.5
S2—C3—H3C	109.5	H12B—C12—H12C	109.5
H3A—C3—H3C	109.5		

### Hydrogen-bond geometry (Å, º)

**Table:**

*D*—H···*A*	*D*—H	H···*A*	*D*···*A*	*D*—H···*A*
C1—H1*C*···I2^i^	0.98	3.03	3.926 (10)	152
C6—H6*B*···I1	0.98	3.05	3.878 (12)	143

Symmetry code: (i) *x*, *y*, *z*−1.
